# Electrochemical Method for Ease Determination of Sodium Diclofenac Trace Levels in Water Using Graphene—Multi-Walled Carbon Nanotubes Paste Electrode

**DOI:** 10.3390/ijerph19010029

**Published:** 2021-12-21

**Authors:** Sorina Motoc, Florica Manea, Anamaria Baciu, Corina Orha, Aniela Pop

**Affiliations:** 1 “Coriolan Drăgulescu” Institute of Chemistry, Romanian Academy, 24 Mihai Viteazu Bvd., 300223 Timisoara, Romania; sorinailies@acad-icht.tm.edu.ro; 2 Department of Applied Chemistry and Engineering of Inorganic Compounds and Environment, Politehnica University of Timisoara, 6 Bv. V. Parvan, 300223 Timisoara, Romania; anamaria.baciu@upt.ro (A.B.); aniela.pop@upt.ro (A.P.); 3 National Condensed Matter Department, Institute for Research and Development in Electrochemistry and Condensed Matter Timisoara, 1 P. Andronescu Street, 300254 Timisoara, Romania; orha.corina@gmail.com

**Keywords:** water quality, water monitoring, sodium diclofenac, graphene, multi-walled carbon nanotubes, paste electrode, electrochemical detection, sorption, preconcentration

## Abstract

Sodium diclofenac (DCF) presence reported in water use cycle at various concentrations including trace levels necessitates continuous development of advanced analytical method for its determination. In this work, ease electrochemical methods for DCF determination based on voltammetric and amperometric techniques were proposed using a simple combination of graphene with multi-walled carbon nanotubes as paste electrode. Integration of the graphene with multi-walled carbon nanotubes enlarged the electroactive surface area of the electrode and implicitly enhanced the electrochemical response for DCF determination. On the basis of the sorption autocatalytic effect manifested at low concentration of DCF, we found that the preconcentration step applied prior to differential-pulsed voltammetry (DPV) and multiple-pulsed amperometry (MPA) allowed for the enhancement of the electroanalytical performance of the DCF electrochemical detections, which were validated by testing in tap water. The lowest limit of detection (LOD) of 1.40 ng·L^−1^ was found using preconcentration prior to DPV under optimized operating conditions, which is better than that reached by other carbon-based electrodes reported in the literature.

## 1. Introduction

Continuous improvement of the environmental quality represents one of the main important objectives of the global research activity efforts. It is obvious that the quality of environment is interconnected to a better control of diseases, drugs, and food quality and safety, and implicit to life quality [1]. A new stage in water management has been marked by the official implementation of the Water Framework Directive on 22 December 2000, which set the ambitious goal of attaining “good status” for each Europe’s water body by 2015 with respect to a well-defined timeline [1,2,3,4]. Effective real-time monitoring based on advanced measurement methods represent the key to understanding and tackling the issue of water quality assurance and, in particular, of water contamination with pharmaceuticals, an actual issue addressed in terms of water quality. Pharmaceuticals administrated to humans or animals are excreted via urine and feces, with 30 to 90% of oral doses generally excreted as active substances [1,2,3,4]. Several variables, including the source and timing of pollution; wastewater treatment plant technology; operation and removal efficiency; the toxicity, degradation, persistence, and mobility properties of the pharmaceuticals; agriculture and veterinary practices; and the sensitivity of the receiving environment and exposure history, are responsible for the concentrations and impacts of pharmaceuticals in the environment [1,2,3,4]. Nonsteroidal anti-inflammatory drugs (NSAIDs) belong to the pharmaceuticals and personal care products (PPCPs) and contain a group of drugs of different chemical composition and therapeutic potentials characterized by a minimum of three common peculiarities: similar basic pharmacological properties and basic mechanism of action and similar adverse effects, as well as those listed in [5]. Diclofenac sodium (DCF) belongs to arylalkanoic acids derivatives; is widely used for the treatment of chronic illnesses and inflammation; and it is purchased without medical prescription, which allows its usage and implicates its high potential to enter in the environment [6]. Its high polarity and hydrophilicity assure easy water transportation, and its presence may be quite high in the groundwater aquifers from the contaminated surface water because it is not significantly retained in the subsoil [5,6,7]. Another important source of pharmaceuticals in the environment is the incomplete treatment of sewage and wastewater. DCF is excreted from the consumer either from residential area or hospital and discharged in sewage and transported into wastewater treatments plants (WWTPs). Due to WWTPs being unable to completely remove or destroy such drugs during wastewater treatment, they are detected in WWTP effluents, which could be further transported into surface water [7,8]. For example, according to O’Flynn et al. (2021), the concentration levels of diclofenac, as well as its main known metabolites (glucuronide, sulfate conjugates) [9], have reached levels of 1.8–181 µg·L^−1^ in WWTP influent, 1.20–24.3 µg·L^−1^ in WWTP effluent, 2.40–140 ng·L^−1^ in drinking water, 185–18,740 ng·L^−1^ in surface water, 3.90–14.00 ng·g^−1^ in sediment-river/streams, and between 4.00 and 1500 ngL^−1^ in sea and ocean surface water. The high-level concentrations detected in water associated with diclofenac met the condition necessary in order to propose diclofenac as a priority substance that requires mandatory testing in surface waters and other water bodies [9].

Taking into account their potential negative impact on the environment and on human health, we argue that the development of the advanced analytical procedures for DCF detection at trace level concentrations is mandatory.

A wide number of non-electrochemical detection methods have been developed for the quantification of DCF, for example, high-performance liquid chromatography (HPLC) [10], gas chromatography–mass spectrometry (GC–MS) [11,12,13], reversed-phase high-performance liquid chromatography (RP-HPLC) [14], liquid–liquid microextraction (LLME) and HPLC analysis [15], and capillary electrophoresis (CE) [16].

Since non-electrochemical detection is usually time-consuming and requires expensive equipment, complex sample pre-treatments that can pose a threat to the environment, and advanced technical expertise, the electrochemical detection offers the advantages of “green analytical chemistry“ through electron involvement as a green reagent, as well as the effective real-time process monitoring [1]. The advantages of electrochemical systems include advanced sensitivity and/or selectivity, a wide linear concentration range, low-cost instrumentation, minimal space, and power consumption [17,18]. In the meantime, the electrochemical techniques have been demonstrated to be useful tools for the development of the detection methods with several advantages, e.g., being easy to operate, cheap, portable, and fast.

In terms of the development of the electrochemical detection methods, the electrode material role is well-known.

Carbon-based electrodes are the most common in terms of electro analysis [5,13,14,15,16,17,18,19,20], and nanostructured carbon has gained an advance in research for the electrochemical detection through its properties and related advantages in the enhancing of the electroanalytic activity [21,22,23,24,25,26,27]. However, in order for a very sensitive electrochemical detection method to bed developed, even nanostructured carbon-based electrodes require further modification to meet the detection performance requirements.

Nanostructured carbon materials (NC) modified with several types of advanced materials, e.g., metal organic framework, copper, chitosan–copper complex, ionic liquids, and gold–platinum bimetallic nanoparticles, have been reported for DCF detection [28,29,30,31,32,33,34,35]. The sensitivities ranging from 0.012 to 1.14 μA·μM^−1^ have been reported for electrochemical detection of DCF using nanostructured carbon-based electrode materials functionalized with copper and platinum [29,30,31,32]. Several combinations of modified electrodes characterized by different detection performances have been developed in our previous reported works [28,33,34,35]. Thus, the sensitivity of 0.015 μA·μM^−1^ was reported for HKUST-1 Metal-Organic Framework modified carbon nanofiber (HKUST-CNF), 42.8 μA·μM^−1^ for Cu-doped zeolite-expanded graphite-epoxy (CUZEGE), and 6.450 μA·μM^−1^ for fullerene–carbon nanofiber paste electrode (F-CNF), with the lowest limits of detection ranging from 0.0009 to 6.15 μM DCF.

All above-presented considerations highlight the necessity of the development of advanced electrochemical methods for detection, with it being the case that the electroanalytical performance is given by the electrode material [33,34,35,36], paying attention to DCF detection.

When the main advantages of carbon paste electrode related to easily renewable and modified surface is taken into account, as well as its low cost, the obtaining of nanostructured carbon paste electrode is facile for its further modification in developing the electrochemical detection of DCF [28,37,38,39]. In fact, the paste composition of the electrode is very easily further modified by simple mixing of carbon with certain oils and modifying component without any template or matrix development. In this study, the integration of graphene from 2D category with the carbon nanotube that belongs to 1D carbon form was considered for the development of the advanced electrochemical detection of DCF at trace levels in water.

## 2. Materials and Methods

### 2.1. Materials

Graphene (GR) was used as received from Sigma-Aldrich (Saint Louis, MO 63103, USA), and multi-walled carbon nanotubes (CNT) were provided by Nanocyl^TM^, Belgium. A total of 1 g·L^−1^ diclofenac sodium salt (DCF) standard stock solution was prepared daily from analytical-grade reagent (Sigma Aldrich) with double-distillated water. A total of 0.100 M Na_2_SO_4_ was freshly prepared from Na_2_SO_4_ of analytical purity (Merck) and used as the supporting electrolyte for the electrode material characterization and application in the detection process.

### 2.2. Obtaining of Working CNT-Based Paste Electrodes

Simple mechanical mixing of certain amount of graphene (GR), multi-walled carbon nanotubes (CNT) within the paraffin oil allowed for the obtaining of electrode paste, as shown in Table 1.

### 2.3. Structural and Morphological Characterization

A scanning electronic microscope (SEM; Inspect S PANalytical model) was used to characterize the morphological structures of CNT and GR-CNT paste electrodes surfaces.

### 2.4. Electrochemical Experiments

The voltammetric techniques, i.e., cyclic voltammetry (CV), differential-pulsed voltammetry (DPV), square-wave voltammetry (SWV), and multiple-pulsed amperometry (MPA) were applied in electrochemical characterization and the amperometric detection tests using an Autolab potentiostat/galvanostat PGSTAT 302N (EcoChemie, Utrecht, the Netherlands), which is controlled by Nova 2.4 software. The classical three-electrode cell, consisting of the working CNT/GR-CNT paste electrodes, a silver/silver chloride reference electrode (Ag/AgCl, KCl 3M) for checking the electrode potential, and a platinum counter electrode to assure the electrical charge transportation, was connected to the potentiostat and controlled by the computer.

The electroactive surface areas of both types of paste electrodes were determined by the cyclic voltammetry (CV) using classical method [40], which is based on an ideal reversible ferri/ferrocyanide system. Cyclic voltammograms (CVs) were recorded in 4 mM K_3_[Fe(CN)_6_] in 1 M KNO_3_ supporting electrolyte at different scan rates at both CNT and GR-CNT paste electrodes, and the apparent diffusion coefficient of the ferri/ferro redox system was determined on the basis of the Randles–Sevcik equation (Equation (1)):

(1) $$I_{p}=2.69\times 10^{5}AD^{1/2}n^{3/2}v^{1/2}C$$

where *A* represents the area of the electrode (cm^2^), *D* the diffusion coefficient of the molecule in solution, *n* the number of electrons participating in the reaction (and is equal to 1), *v* the scan rate (*V* s^−1^), and *C* the concentration of the probe molecule in the solution.

To elucidate several mechanistic aspects of the oxidation/reduction processes of DCF onto the electrode surface, we investigated the influence of the scan rate on CV shapes at various scan rates between 0.010 and 0.200 *V*·s^−1^, and the linear dependence of the oxidation peak current was checked.

The lowest limit of detection (LOD) and the limit of the quantification (LQ) were determined on the basis of the equations of LOD = 3 SD/m and LQ = 10 SD/m, where SD is the standard deviation of three blanks and m is the slope of the analytical plots [41]. The reproducibility of the electrodes using the above-mentioned technique was evaluated by the relative standard deviation (RSD) for three replicates measurements of DCF concentration.

## 3. Results and Discussions

### 3.1. Morphological and Electrochemical Characterization

**Figure 1:**
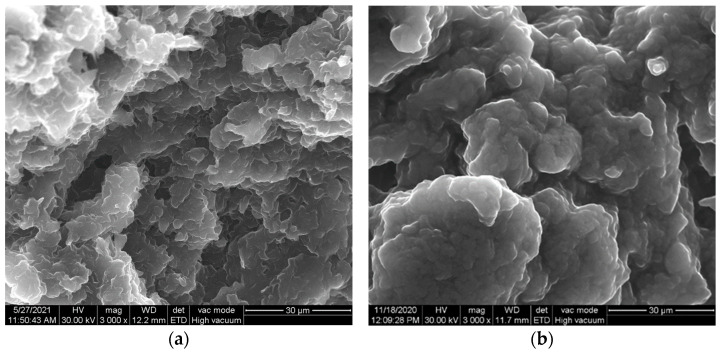
SEM images of paraffin oil-based carbon paste: (**a**) CNT and (**b**) GR-CNT.

Figure 1a,b shows the SEM images of the CNT and GR-CNT pastes in the paraffin oil. The graphene (GR) integration within multi-walled carbon nanotubes paste composition modified the morphostructural characteristics of the structural conformations of CNT (1D) and GR (2D). GR presence covered the structure of CNT, and it was significantly manifested through a more porous morphology.

The electroactive electrode areas were calculated by comparing the apparent diffusion coefficient value determined by classical method with the theoretical diffusion coefficient value of 6.70·10^−6^ cm^2^·s^−1^ reported in the literature data [41], and the results are presented in Table 2. The electroactive surface areas determined for both CNT and GR-CNT paste electrodes were higher in comparison with the geometrical ones; GR-CNT exhibited the best electroactive area because the content of carbon was higher and the specific surface area was higher for GR than for CNT [42].

**Figure 2:**
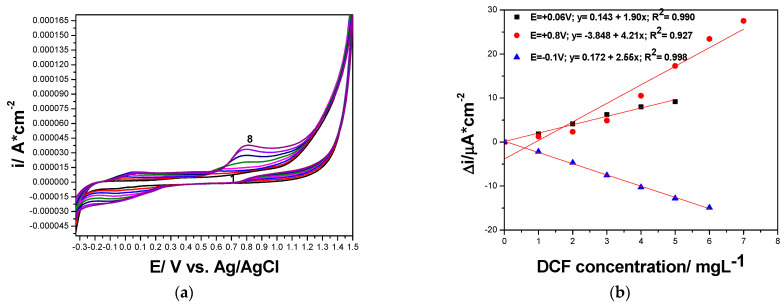
(**a**) Cyclic voltammograms recorded at CNT paste electrode with the scan rate of 0.050 V·s^−1^ in 0.100 M Na_2_SO_4_ supporting electrolyte (curve 1), and DCF concentrations ranged from 1.00 to 7.00 mg·L^−1^ (curve 2–8). (**b**) Calibrations plots of peak current vs. DCF concentrations at the potential value: E = +0.060 V vs. Ag/AgCl (anodic), E = +0.800 V vs. Ag/AgCl (anodic), and E = −0.100 V vs. Ag/AgCl (cathodic).

**Figure 3:**
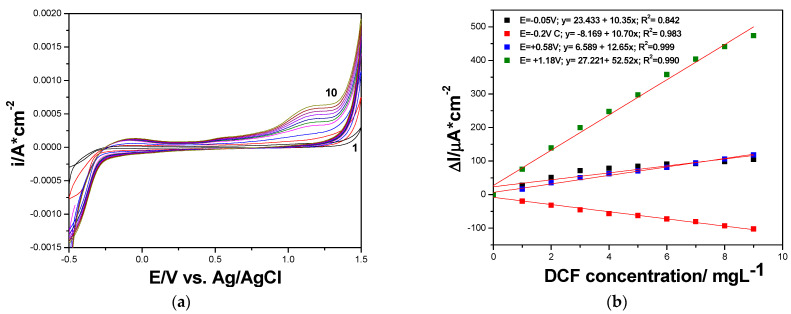
(**a**) Cyclic voltammograms recorded at GR-CNT paste electrode with the scan rate of 0.050 V·s^−1^ in 0.100 M Na_2_SO_4_ supporting electrolyte (curve 1), and DCF concentrations ranged from 1.00 to 9.00 mg·L^−1^ (curve 2–10). (**b**) Calibrations plots of peak current vs. DCF concentrations at the potential value: E = −0.050 V vs. Ag/AgCl (anodic), E = +0.580 V vs. Ag/AgCl (anodic), E = +1.180 V vs. Ag/AgCl (anodic), and E = −0.200 V vs. Ag/AgCl (cathodic).

Both CNT and GR-CNT paste electrodes were comparatively electrochemically characterized in 0.100 M Na_2_SO_4_ supporting electrolyte and in the presence of different DCF concentrations by CV, and several differences between the shapes of the two CV series were found, which are presented in Figure 2a,b and Figure 3a,b. CVs recorded only in 0.100 M Na_2_SO_4_ supporting electrolyte without DCF addition showed that GR incorporation within the CNT-based composition enhanced the background current that is attributed to the capacitive component due to more significant double-layer characteristics, and the polarization effect was also evidenced, which enlarged the potential window for GR-CNT paste electrode. This behavior is linked to the structural characteristics of GR that belong to 2D class in comparison with CNT from 1 D class. The electrochemical oxidation of DCF started early, quite within the cathodic range (−0.200 and −0.100 V vs. Ag/AgCl for GR-CNT and for CNT paste electrode) and continued over the whole anodic range until the oxygen evolution potential (+1.30 V and +1.05 V vs. Ag/AgCl for GR-CNT and for CNT paste electrode). Two anodic peaks (+0.060 and +0.800 V vs. Ag/AgCl) attributed to DCF electrooxidation were evidenced for CNT paste electrode, while for GR-CNT, three anodic peaks appeared (−0.050, +0.530, and +1.180 V vs. Ag/AgCl), which showed that DCF electrooxidation took place in two steps onto CNT paste electrode and in three steps for GR-CNT paste electrode. During reverse scanning through CV, we observed that for both CNT-based paste electrodes, a cathodic peak corresponding to the first anodic one more obviously appeared at potential value of −0.100 V vs. Ag/AgCl for CNT paste electrode compared with GR-CNT paste electrode at potential value of −0.200 V vs. Ag/AgCl. This peak showed a possible quasi-reversible oxidation-reduction step of DCF after checking the difference between anodic and cathodic peak potential (0.150 V ≠ 0.059 V/n, where n is number of electrons, which is a component characteristic to the ideal reversible system), and the ratio between anodic and cathodic current height (/ia/ic/ ≠ 1), which is other component characteristic for the ideal reversible system). All dependences of the current densities recorded at both anodic and cathodic peaks potential values vs. DCF concentrations were linear (Figure 2b,d), characterized by different slopes, which informed us about the different sensitivities for DCF determination by CV techniques with both CNT paste electrodes.

An overview of results regarding the sensitivities and the detection potentials of DCF on both CNT and GR-CNT paste electrodes are gathered in Table 3.

Considering the high value of Y intercept of the calibration plots reached for GR-CNT paste electrode, we tested CV for the range of 10 times lower DCF concentrations (0.100 to 1.20 mg·L^−1^), finding that the sensitivity was better (Figure S1). For the DCF concentrations ranging between 0.01 and 0.10 mg·L^−1^, no good correlation of the calibration plots was achieved (R^2^ = 0.865), probably due to the electrode surface-controlled process that modified the electrochemical response. Moreover, it was very interesting that for lower DCF concentration, another oxidation peak appeared at the potential value of + 1.350 V vs. Ag/AgCl, which was attributed to a further step of the DCF oxidation on the electrode surface.

It can be concluded that GR integrated within CNT paste electrode composition exhibited better features for the DCF detection, and this electrode was selected for further investigation of the DCF detection optimization. Considering the effect of the DCF concentration range on the CV shape and for the deep electrochemical characterization of DCF electrooxidation on GR-CNT electrode, we investigated the influence of the scan rate on CV shape separately for different DCF concentration ranges (0.005, 0.050, and 0.500 mg·L^−1^ DCF), and the dependences of the each peak current vs. the scan rate are gathered in Table 4. There were several differences related to the number of anodic oxidation peaks (oxidation steps) and the non-linear/linear dependence of the current vs. the square rate. For very low DCF concentrations (5.00 µg·L^−1^), non-linear dependence of the peak current vs. the square root of the scan rate was found at the potential value of −0.020 V·s^−1^, probably due to the surface-controlled process occurring. Moreover, between the phenyl rings of DCF and the carbon nanotubes and graphene, the interaction π–π should appear, which changes the voltammetric profile [43]. For higher DCF concentration, at which all anodic peak currents depend linearly on the square root of the scan rate, the processes were controlled by the diffusion steps that are desired for the electrochemical detection. Moreover, for the intermediary range of the DCF concentrations, another anodic peak corresponding to the further DCF oxidation appeared very close to the oxygen evolution at the potential value of +1.350 V vs. Ag/AgCl.

**Figure 4:**
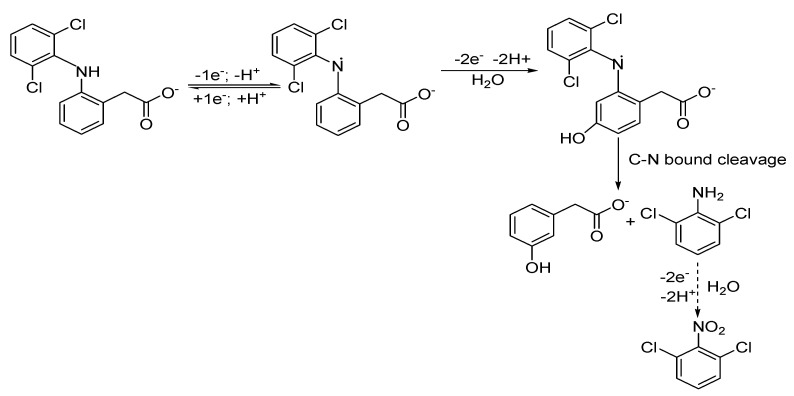
A proposed mechanism of DCF oxidation.

It is clear that the oxidation mechanism is influenced by the DCF concentrations, and considering the DCF oxidation mechanisms proposed in the literature [44,45], we proposed a probable overall complex mechanism of the direct oxidation of DCF on the GR-CNT paste electrode surface. The mechanism considers a complex EE-ECE model and the sorption autocatalytic effect [46], in which the first electrochemical stage (E) is reversible and the other electrochemical and chemical (EC) stages are irreversible (see Figure 4).

DCF began to be reversibly oxidized at quite a negative potential value of −0.05 V vs. Ag/AgCl, and DCF radicals were generated, which were further irreversibly oxidized onto the electrode surface to 5-hydroxidiclofenac radical by a loss of 2e^−^, with 2H^+^ giving rise to an oxidation peak at about +0.580 V [47]. During further scanning in the anodic direction at the potential value of about +1.150 V vs. Ag/AgCl, 2,6 dichloro-aniline and 2-(2-hydroxyphenyl) acetic acid were formed through the C-N bound cleavage, in accordance with the literature [48]. At medium DCF concentrations, the anodic peak that appeared at higher potential value near to the oxygen evolution could be attributed to the further oxidation of 2,6 dichloro-aniline to 2,6 dichloro-nitrobenzene.

### 3.2. Development of DCF Electrochemical Detection

**Figure 5:**
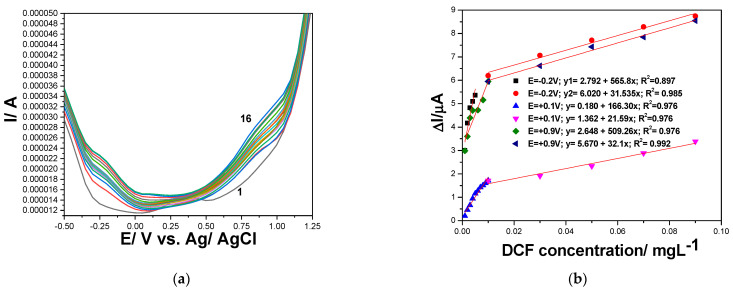
(**a**) Differential-pulsed voltammograms at GR-CNT electrode in 0.100 M Na_2_SO_4_ supporting electrolyte (curve 1) and in the presence of different DCF concentrations: curves 2–16: 0.001–0.110 mgL^−1^ DCF; 50 mV step potential; 200 mV modulation amplitude, 100 mV·s^−1^ potential scan rate; potential range: −0.500 to +1.50 V vs. Ag/AgCl. (**b**) Calibration plots of the currents recorded at E = −0.200 V; +0.100 V and +0.900 V vs. Ag/AgCl, versus DCF concentrations.

Differential-pulsed and square-wave voltammetry (DPV and SWV) were tested to develop the highest performant and respective, fast voltammetric detection scheme for DCF determination, and multiple-pulsed amperometry (MPA) for the amperometric detection of DCF. DPV parameters were evaluated within the range from 25 to 250 mV for modulation amplitude (MA) and from 10 to 100 mV for the step potential (SP) with the MA/SP ratio ranging from 8:1 to 1:1, which allowed for the obtaining of the stable response of the electrode. The optimized parameters were 200 mV of MA and 50 mV of SP at the scan rate of 100 mVs^−1^. Figure 5a depicts the DPV measurements of DCF oxidation at various concentrations from 1.00 to 110 µg·L^−1^ DCF, and the corresponding analytical curve is presented in Figure 5b. A linear dependence with a good correlation coefficient was verified for this DCF concentration range, which was extended up to 1.00 mg·L^−1^ DCF. In comparison with CV, the values of the oxidation peak potentials were shifted to less positive potentials, and great improvement of the sensitivities was achieved (see Table 5). The lowest limit of detection (LOD) for DCF determination was calculated for each DCF oxidation peak on the basis of the sensitivity determined from the slope of the analytical curve. The relative standard deviation (RSD) determined for three replicates were lower than 1.00%, and the reproducibility of the electrode surface determined by using new batch of similar composition paste was 5.00%.

**Figure 6:**
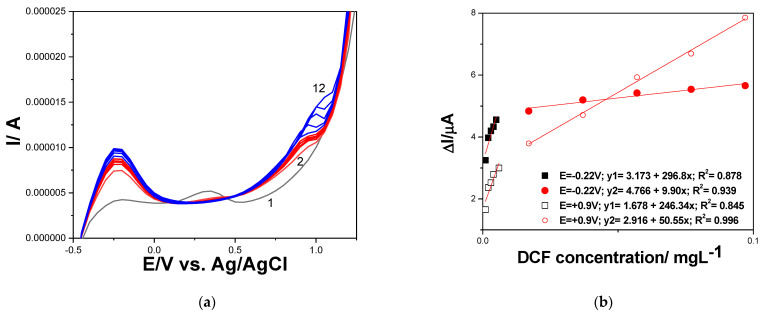
(**a**) Square-wave voltammograms recorded on GR-CNT electrode in 0.100 M Na_2_SO_4_ supporting electrolyte (curve 1) and in the presence of various DCF concentrations: curves 2–11: 0.001–0.010 mg·L^−1^ DCF; step potential of 50 mV; modulation amplitude of 200 mV, frequency of 2 Hz, potential scan rate: 100 mV·s^−1^; potential range: −0.500 to +1.50 V vs. Ag/AgCl. (**b**) Calibration plots of the currents recorded at E = −0.220 (filled symbols), and E = +0.900 V (unfilled symbols) vs. Ag/ AgCl, versus DCF concentration.

SWV was also tested for DCF detection due to its fast response. The optimized operating conditions found for DPV were tested at different frequencies, and the best performance was achieved for the frequency of 2 Hz at the scan rate of 100 mVs^−1^. Figure 6a shows the SWV measurements of DCF oxidation at various concentrations from 1 to 10 µgL^−1^ DCF, and the corresponding calibration plots are presented in Figure 6b. The sensitivities were lower in comparison with those reached by DPV, and RSD was slight higher. The limit of detection was also worse for SWV vs. DPV (see Table 5).

### 3.3. Preconcentration Step Prior to DPV Detection

**Figure 7:**
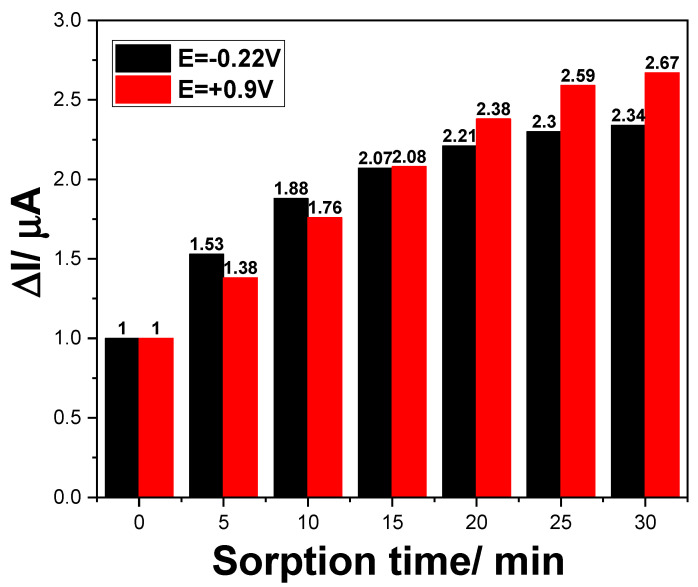
Voltammetric signals achieved for 0.500 µg·L^−1^ DCF by DPV recorded on GR-CNT paste electrode as a function of the sorption time in the preconcentration step prior to detection recorded at E = −0.220 V and +0.900 V vs. Ag/AgCl.

For further improvement of the optimized DPV detection of DCF, considering the sorption property of the CNT and, especially, GR, towards DCF, which should have negatively influenced the higher and medium concentrations of DCF, we considered the development of a preconcentration step-based detection method to achieve DCF detection at trace concentration levels. Different times of maintaining the electrode immersed in 0.500 µg·L^−1^ DCF and 0.100 M Na_2_SO_4_ supporting electrolyte at open circuit potential (OCP) were applied to study the sorption effect on the electrode response. The DPVs recorded after different sorption times in the same 0.500 µg·L^−1^ DCF are presented in Figure 7, and the preconcentration factor of about 2.50 times was achieved after 25 min, which was selected as optimum sorption time. Applying the sorption time of 25 min made it possible to obtain good voltammetric response for detection of DCF ranging from 10 to 60 ng·L^−1^ (see Figure S2), and much better sensitivity and LOD (1.40 ng·L^−1^ for −0.210 V vs. Ag/AgCl and 2.30 ng·L^−1^ for +0.900 V vs. Ag/AgCl) were reached.

It is well known that a very performant option of the amperometric detection is multiple-pulsed amperometry (MPA), which should be proposed on the basis of the CV result considered as a reference. Considering the large potential window in which DCF is oxidized in several steps, we considered two potential values as detection ones, and one more for advanced oxidation within the oxygen range evolution to assure in situ cleaning of the electrode surface. In addition, the negative potential value of −0.400 V vs. Ag/AgCl was considered for the electrode surface refresh and renewal. Different multiple-pulsed amperometric schemes were considered in terms of the pulse order and potential value, but the optimized MPA schemes consisted of

E1 = −0.400 V vs. Ag/AgCl for 0.10 s for renewing electrode surface;
E2 = −0.020 V vs. Ag/AgCl for 0.05 s, representing the first step of DCF oxidation considered as the first detection potential;
E3 = +0.600 V vs. Ag/AgCl for 0.05 s, considered the second detection potential due to second step of DCF oxidation;
E4 = +1.500 V vs. Ag/AgCl for 0.10 s, applied to assure in situ electrode surface cleaning based on concomitant slight rate of oxygen evolution.

**Figure 8:**
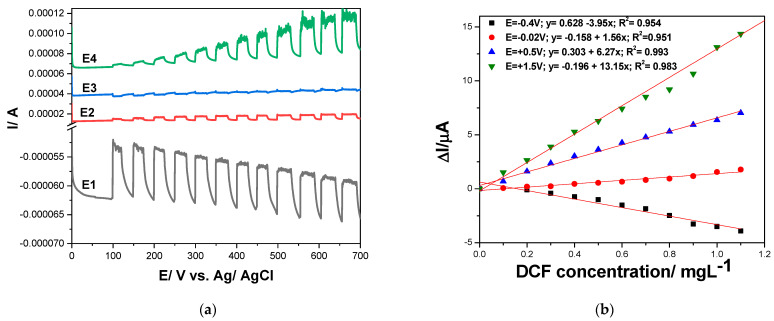
(**a**) Multiple-pulsed amperograms recorded by GR-CNT electrode in 0.1 M Na_2_SO_4_ supporting electrolyte and 0.100 mg·L^−1^ DCF added consecutively and continuously, recorded at E1 = −0.400 V vs. Ag/AgCl, E2 = −0.020 V vs. Ag/AgCl, E3 = +0.500 V vs. Ag/AgCl, and E4 = +1.50 V vs. Ag/AgCl. (**b**) Calibration plots of the currents recorded at E = −0.400 V vs. Ag/AgCl, E = −0.020 V vs. Ag/AgCl, E = +0.500 V vs. Ag/AgCl, and E = +1.50 V vs. Ag/AgCl versus diclofenac concentrations.

The amperograms recorded by MPA under the above-presented operating conditions are presented in Figure 8a, and the linear dependences of the current vs. DCF concentrations at all applied potentials are shown in Figure 8b. As was expected, a cathodic response was found at −0.400 V vs. Ag/AgCl, while for other potential values, the anodic responses were achieved. It can be easily noticed that the electroanalytical performance related to both the lowest limit of detection (LOD) and the sensitivity were better for multiple-pulsed amperometric technique in comparison with CV results. A further enhancement of the sensitivity and the LOD was achieved by applying the preconcentration step for 25 min prior the multiple-pulsed amperograms recording, when the preconcentration factor of about 26.5 was found (see Table 5).

### 3.4. Testing Preconcentration Step-Based Multiple-Pulsed Amperometry in Tap Water

**Figure 9:**
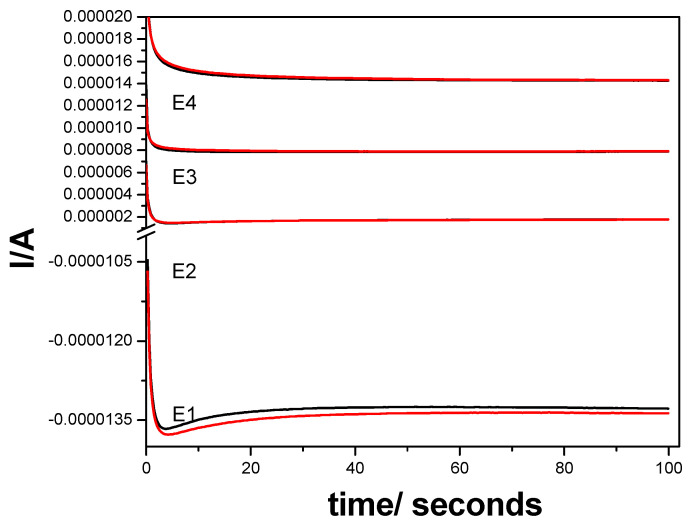
Multiple-pulsed amperograms recorded in tap water by applying a preconcentration step of 25 min GR-CNT electrode in 0.100 M Na_2_SO_4_ supporting electrolyte and adding 0.500 µg·L^−1^ DCF, recorded at E1 = −0.400 V vs. Ag/AgCl, E2 = −0.020 V vs. Ag/AgCl, E3 = +0.500 V vs. Ag/AgCl, and E4 = +1.500 V vs. Ag/AgCl.

The tap water spiked with DCF concentration of 0.500 µgL^−1^ was prepared and analyzed with the GR-CNT paste electrode using the optimized preconcentration step-based multiple-pulsed amperometry, and the result presented in Figure 9 shows that the recovery degree of 104.9% was found. To assure the preconcentration step, one must record the whole multiple-pulsed amperometry for each DCF concentration, and the continuous concentration adding is not appropriate. Finally, the results obtained by DPV and MPA for the DCF concentrations of 0.50, 1.00, and 1.50 mgL^−1^ were compared with those obtained by means of a conventional spectrophotometrical method. The results of both methods were similar, showing a good accuracy of the DCF detection using GR-CNT with both DPV and MPA techniques.

Repeatability of the preconcentration-based MPA detection procedure with GR-CNT was evaluated by comparing the results of the determination of a 0.50 µgL^−1^ DCF during the one week, with the relative standard deviation less than 5.00% demonstrating a good repeatability of the proposed detection method.

The electroanalytical performances for DCF detection obtained with GR-CNT electrode using both voltammetric and amperometric techniques in 0.100 M Na_2_SO_4_ supporting electrolyte are gathered in Table 5. As was expected, the optimization of DPV technique allowed for the enhancement of the sensitivity and the lowest limit of detection for DCF determination. Moreover, multiple-pulsed amperometry tested under optimized conditions led to good electroanalytical performance, quite better in comparison with the CV one. In addition, the sorption capacity of GR-CNT electrode surface was exploited in a positive way to accumulate the trace levels of DCF onto the electrode surface, assuring a further improvement of the electroanalytical performance for DCF determination in the aqueous solution.

By comparison with other carbon-based electrodes, which have been reported for the development of the electrochemical methods for DCF determination, GR-CNT paste electrode showed the best limit of detection (see Table 6), which made this electrode be considered for the electrochemical detection of DCF trace levels.

It is obviously the case that besides the electrode composition, the electrochemical technique exhibits a main role to develop the advanced electrochemical detection for DCF. Taking into account the advantages and disadvantages of voltammetric and amperometric techniques, as well as the detection purpose with the respect of the water type and matrix, we found that GR-CNT shows a great utility for practical application in the development of the screening method for the determination of DCF in any type of water body (WWTP effluent, surface water, and drinking water).

## 4. Conclusions

Simple integration of graphene within multi-walled carbon nanotube paste electrode (GR-CNT) led to a stable and higher electrochemical response in 0.1 M Na_2_SO_4_ supporting electrolyte due to larger electroactive surface area in comparison with multi-walled carbon nanotubes paste electrode (CNT). The comparative electrochemical signal of the diclofenac onto GR-CNT and CNT electrodes was characterized by cyclic voltammetry, and better voltammetric response was achieved for GR-CNT electrode. A very complex mechanism of the DCF oxidation on GR-CNT electrode was found by a systematic study of the scan rate influence on the CV shapes related to the DCF concentration ranges. Sorption autocatalytic effect found at low DCF concentration allowed for the enhancement of the electroanalytical performances of both advanced voltammetric and amperometruic techniques optimized for DCF detection. Applying the optimized sorption time of 25 min prior to the differential-pulsed voltammetry under operating conditions of 200 mV MA and 50 mV SP at the scan rate of 100 mVs^−1^, we achieved the lowest limit of detection (LOD) of 1.40 ng·L^−1^, which made the method suitable for DCF detection at trace levels. Moreover, the same preconcentration step was applied prior to optimized multiple-pulsed amperometry, which was validated in real tap water for DCF detection. Selection of voltammetric and/or amperometric techniques for DCF detection with GR-CNT will consider the practical purpose with the respect of the water type and matrix. GR-CNT shows a great utility for practical application in the development of the screening monitoring method for the determination of DCF in water usage cycle (WWTP effluent, surface water, and drinking water).

## Figures and Tables

**Table 1:** The composition of the working paste electrodes.

Weight Ratio, %			
Paste Electrode Type	Carbon Nanotubes (CNT)	Graphene (GR)	Paraffin Oil
CNT	1	-	3
GR-CNT	1	1	3.5

- none added.

**Table 2:** Electroactive surface areas in comparison with geometrical ones.

Electrode	Geometrical Surface Area/cm^2^	Electroactive Surface Area/cm^2^	Electroactive/Geometrical Surface Area Ratio
CNT	0.0765	0.117	1.53
GR-CNT	0.0176	0.038	2.16

Carbon Nanotubes (CNT). Graphene (GR).

**Table 3:** Sensitivities and detection potential values for DCF determination with CNT and GR-CNT paste electrodes.

Electrode Type	Potential Range	E_det_ (V vs. Ag/AgCl)	Sensitivity (µA/cm^2^·mg·L^−1^)
CNT	Anodic	+0.060	1.90
	Anodic	+0.800	4.21
	Cathodic	−0.100	2.55
GR-CNT	Anodic	−0.050	10.3
	Anodic	+0.580	12.7
	Anodic	+1.180	52.5
	Cathodic	−0.200	10.7

**Table 4:** Equations of the anodic peaks currents vs. the square root of the scan rates for different DCF concentration ranges (0.005, 0.050, and 0.500 mg·L^−1^ DCF).

DCF Concentration, mg·L^−1^	E = −0.050 V vs. Ag/AgCl	E = +0.580 V vs. Ag/AgCl	E = +1.150 V vs. Ag/AgCl	E = +1.350 V vs. Ag/AgCl
0.500	y_1_ = −0.934 + 4.17; R^2^ = 0.983	y_2_ = −0.445 + 6.30×; R^2^ = 0.976	y_3_ = −0.383 + 18.45×; R^2^ = 0.994	- **
0.050	y_1_ = 0.162 + 2.82×; R^2^ = 0.952	y_2_= −0.292 + 0.081×; R^2^ = 0.967	y_3_ = 0.778 + 21.55×; R^2^ = 0.995	Y_4_ = 1.791 + 45.89×; R^2^ = 0.985
0.005	- *	y_2_ = −0.191 + 2.76×; R^2^ = 0.992	y_2_= −0.795 + 10.52×; R^2^ = 0.990	- **

* no linear dependence; ** no oxidation peak appeared. Sodium diclofenac (DCF).

**Table 5:** The electroanalytical parameters for diclofenac detection with GR-CNT paste electrode in 0.100 M Na_2_SO_4_ supporting electrolyte.

Technique	Detection Potential/ V vs. Ag/AgCl	Sensitivity/ µA·mgL^−1^	Correlation Coefficient/R^2^	LOD ^[a]^/ mg·L^−1^	LQ ^[a]^/ mg·L^−1^	RSD ^[b]^ (%)
CV	−0.02	0.225	0.956	0.53	1.78	8.69
	−0.02 (C *)	0.066	0.962	2.02	6.76	7.39
	+0.53	0.378	0.982	0.43	1.44	9.14
	+1.15	6.38	0.976	0.02	0.08	3.31
	+1.35	9.40	0.998	0.01	0.05	1.31
DPV	+0.9	509.26	0.976	1.7 × 10^−4^	5.1 × 10^−4^	0.14
Preconc. DPV	−0.21	31,400	0.954	1.4 × 10^−6^	4.6 × 10^−6^	0.22
	+0.9	51,000	0.946	2.3 × 10^−6^	7.8 × 10^−6^	0.35
SWV	+0.9	50.55	0.996	7.4 × 10^−3^	2.1 × 10^−2^	1.6
MPA	+1.5 V	13.15	0.983	2.8 × 10^−2^	9.4 × 10^−2^	0.18
Preconc. MPA	+1.5 V	347.32	0.968	5.9 × 10^−4^	1.97 × 10^−3^	0.48

^[a]^ LOD—the lowest limit of detection, LQ—limit of quantification. ^[b]^ RSD—relative standard deviation. * cathodic signal. cyclic voltammetry (CV). differential-pulsed voltammetry (DPV). square-wave voltammetry (SWV). multiple-pulsed amperometry (MPA).

**Table 6:** Comparative performances for electrochemical sensing of diclofenac of GR-CNT paste electrode and carbon-based electrodes reported in the literature.

Method	Electrode	Modifier	LOD (ng·L^−1^)	Matrix	Ref.
DPAdSV	SPCE	COOH-CNT	8.30	River water	[49]
Preconc/SWV	CNF	Fullerene	265	Water	[14]
SWV	CPE	CNT-vinylferrocene	590 × 10^3^	Tablets and urine	[50]
DPV	GCE	CNT/Cu(OH)2/1-EM1MPF6	11.80 × 10^3^	Ampoule, tablets, blood serum, fish serum, seawater	[29]
DPV	GCE	PDDA-Gr	179.6 × 10^3^	Tablet, lake water	[51]
SWV	GCE	CNT-CHT	6.20 × 10^3^	Tablets, urine	[30]
Prec. DPV	CNT	Graphene	1.40	Tap water	This work
Prec/MPA	CNT	Graphene	590	Tap water	This work

## Supplementary Materials

The following supporting information can be downloaded at: https://www.mdpi.com/article/10.3390/ijerph19010029/s1, Figure S1: (a) CVs recorded at GR-CNT paste electrode with the scan rate of 0.050 V·s^−1^ in 0.100 M Na_2_SO_4_ supporting electrolyte (curve 1) and DCF concentrations ranging from 0.100 to 1.20 mgL^−1^ (curve 2–6). (b) Calibration plots of peak current vs. DCF concentrations at the potential value: E = −0.050 V vs. Ag/AgCl (anodic), E = +0.580 V vs. Ag/AgCl (anodic), E = +1.180 V vs. Ag/AgCl (anodic), E = +1.350 V vs. Ag/AgCl (anodic), and E = −0.200 V vs. Ag/AgCl (cathodic). Figure S2: (a) Differential-pulsed voltammograms recorded on GR-CNT paste electrode in 0.100 M Na_2_SO_4_ supporting electrolyte (curve 1) with optimum accumulation time of 25 min in the presence of different DCF concentrations: 10–60 ng·L^−1^ (curves 2–7); step potential of 50 mV, modulation amplitude of 200 mV, and a scan rate of 100 mV·s^−1^, potential range: −0.500 V to +1.500 V vs. Ag/AgCl. (b) Calibration plots of peak currents vs. DCF concentrations at the potential value: E = −0.210 V vs. Ag/AgCl, E = +0.900 V vs. Ag/AgCl.

## References

[1] Nannou C.I., Kosma C.I., Albanis T.A. (2015). Occurrence of pharmaceuticals in surface waters: Analytical method development and environmental risk assessment. Int. J. Environ. Anal. Chem..

[2] Vasquez M.I., Lambrianides A., Schneider M., Kümmerer K., Fatta-Kassinos D. (2014). Environmental side effects of pharmaceutical cocktails: What we know and what we should know. J. Hazard. Mater..

[3] Musson S.E., Townsend T.G. (2009). Pharmaceutical compound content of municipal solid waste. J. Hazard. Mater..

[4] Aus der Beek T., Weber F.A., Bergmann A., Hickmann S., Ebert I., Hein A., Küster A. (2016). Pharmaceuticals in the environment-global occurrences and perspectives: Pharmaceuticals in the global environment. Environ. Toxicol. Chem..

[5] Amos Sibeko P., Naicker D., Mdluli P.S., Madikizela L.M. (2019). Naproxen, ibuprofen, and diclofenac residues in river water, sediments and Eichhornia crassipes of Mbokodweni river in South Africa: An initial screening. Environ. Forensics.

[6] Gouda A.A., Kotb El-Sayed M.I., Amin A.S., El Sheikh R. (2013). Spectrophotometric and spectrofluorometric methods for the determination of non-steroidal anti-inflammatory drugs: A review. Arab. J. Chem..

[7] Madikizela L.M., Chimuka L. (2016). Determination of ibuprofen, naproxen and diclofenac in aqueous samples using a multi-template molecularly imprinted polymer as selective adsorbent for solid-phase extraction. J. Pharm. Biomed..

[8] Petrie B., Barden R., Kasprzyk-Hordern B. (2015). A review on emerging contaminants in wastewaters and the environment: Current knowledge, understudied areas and recommendations for future monitoring. Water Res..

[9] O’Flynn D., Lawler J., Yusuf A., Parle-McDermott A., Harold D., Mc Cloughlin T., Holland L., Regan F., White B. (2021). A review of pharmaceutical occurrence and pathways in the aquatic environment in the context of a changing climate and the COVID-19 pandemic. Anal. Methods.

[10] Madikizela L.M., Chimuka L. (2017). Simultaneous determination of naproxen, ibuprofen and diclofenac in wastewater using solid-phase extraction with high performance liquid chromatography. Water SA.

[11] Samaras V.G., Thomaidis N.S., Stasinakis A.S., Gatidou G., Lekkas T.D. (2010). Determination of selected non-steroidal anti-inflammatory drugs in wastewater by gas chromatography-mass spectrometry. Int. J. Environ. Anal. Chem..

[12] Maurer H.H., Tauvel F.X., Kraemer T. (2001). Screening procedure for detection of non-steroidal anti-inflammatory drugs and their metabolites in urine as part of a systematic toxicological analysis procedure for acidic drugs and poisons by gas chromatography- mass spectrometry after extractive methylation. J. Anal. Toxicol..

[13] Wolecki D., Caban M., Pazdro K., Mulkiewicz E., Stepnowski P., Kumirska J. (2019). Simultaneous determination of non-steroidal anti-inflammatory drugs and natural estrogens in the mussels Mytilus edulis trossulus. Talanta.

[14] Patel P.N., Samanthula G., Shrigod V., Modh S.C., Chaudhari J.R. (2013). RP-HPLC method for determination of several NSAIDs and their combination drugs. Chromatogr. Res. Int..

[15] Park S.Y., Myung S.-W. (2015). Simultaneous determination of nonsteroidal anti-inflammatory drugs in aqueous samples using dispersive liquid-liquid microextraction and HPLC analysis. Bull. Korean Chem. Soc. B.

[16] Quek N.M., Law W.S., Lau H.F., Zhao J.H., Hauser P.C., Li S.F.Y. (2008). Determination of pharmaceuticals classified as emerging pollutants using capillary electrophoresis with capacitively coupled contactless conductivity detection. Electrophoresis.

[17] Saleh T.A. (2016). Nanomaterials for pharmaceuticals determination. Bioenergetics.

[18] Kurbanoglu S., Ozkan S.A. (2018). Electrochemical carbon based nanosensors: A promising tool in pharmaceutical and biomedical analysis. J. Pharm. Biomed. Anal..

[19] Hu J., Zhang Z. (2020). Application of electrochemical sensors based on carbon nanomaterials for detection of flavonoids. Nanomaterials.

[20] Torrinha A., Oliveira T.M.B.F., Ribeiro F.W.P., Correia A.N., Lima-Neto P., Morais S. (2020). Application of nanostructured carbon-based electrochemical (Bio)sensors for screening of emerging pharmaceutical pollutants in waters and aquatic species: A review. Nanomaterials.

[21] Yang X., Feng B., He X., Li F., Ding Y., Fei J. (2013). Carbon nanomaterial based electrochemical sensors for biogenic amines. Microchim. Acta.

[22] Power A.C., Gorey B., Chandra S., Chapman J. (2018). Carbon nanomaterials and their application to electrochemical sensors: A review. Nanotechnol. Rev..

[23] Xie F., Yang M., Jiang M., Huang X.-J., Liu W.-Q., Xie P.-H. (2019). Carbon based nanomaterials—A promising electrochemical sensor toward persistent toxic substance. Trends Analyt. Chem..

[24] Siqueira J.R., de Oliveira O.N. (2017). Nanostructures.

[25] Tiwari J.N., Vij V., Kemp K.C., Kim K.S. (2015). Engineered carbon-nanomaterial-based electrochemical sensors for biomolecules. ACS Nano.

[26] Manea F. (2017). Modern Electrochemical Methods in Nano, Surface and Corrosion Science.

[27] Pan M., Yin Z., Liu K., Du X., Liu H., Wang S. (2019). Carbon-based nanomaterials in sensors for food safety. Nanomaterials.

[28] Motoc S., Manea F., Orha C., Pop A. (2019). Enhanced electrochemical response of diclofenac at a fullerene-carbon nanofiber paste electrode. Sensors.

[29] Arvand M., Gholizadeh T.M., Zanjanchi M.A. (2012). MWCNTs/Cu(OH)_2_ nanoparticles/IL nanocomposite modified glassy carbon electrode as a voltammetric sensor for determination of the non-steroidal anti-inflammatory drug diclofenac. Mater. Sci. Eng. C.

[30] Shalauddin M., Akhter S., Bagheri S., Karim M.S.A., Adib Kadri N., Basirun W.J. (2017). Immobilized copper ions on MWCNTS-Chitosan thin film: Enhanced amperometric sensor for electrochemical determination of diclofenac sodium in aqueous solution. Int. J. Hydrogen Energy.

[31] Ensafi A.A., Izadi M., Karimi-Maleh H. (2012). Sensitive voltammetric determination of diclofenac using room-temperature ionic liquid-modified carbon nanotubes paste electrode. Ionics.

[32] Eteya M.M., Rounaghi G.H., Deiminiat B. (2018). Fabrication of a new electrochemical sensor based on Au Pt bimetallic nanoparticles decorated multi-walled carbon nanotubes for determination of diclofenac. Microchem. J..

[33] Motoc S., Manea F., Iacob A., Martinez-Joaristi A., Gascon J., Pop A., Schoonman J. (2016). Electrochemical selective and simultaneous detection of diclofenac and ibuprofen in aqueous solution using HKUST-1 metal-organic framework-carbon nanofiber composite elect. Sensors.

[34] Ihos M., Remes A., Manea F. (2012). Electrochemical determination of diclofenac using boron-doped diamond electrode. J. Environ. Prot. Ecol..

[35] Manea F., Ihos M., Remes A., Burtica G., Schoonman J. (2010). Electrochemical determination of diclofenac sodium in aqueous Solution on cu-doped zeolite-expanded graphite-epoxy electrode. Electroanalysis.

[36] Boumya W., Taoufik N., Achak M., Bessbousse H., Elhalil A., Barka N. (2021). Electrochemical sensors and biosensors for the determination of diclofenac in pharmaceutical, biological and water samples. Talanta Open.

[37] Voda R.D., Negrea S., Păcurariu C., Surdu A., Ianculescu A., Pop A., Manea F. (2021). CuBi2O4 synthesis, characterization, and application in sensitive amperometric/voltammetric detection of amoxicillin in aqueous solutions. Nanomaterials.

[38] Motoc S., Cretu C., Costisor O., Baciu A., Manea F., Szerb E.I. (2019). Cu(I) coordination complex precursor for randomized CuOx microarray loaded on carbon nanofiber with excellent electrocatalytic performance for electrochemical glucose detection. Sensors.

[39] Motoc S., Schinteie B., Pop A., Negrea S., Cretu C., Szerb E.I., Manea F. (2021). Graphene quantum dots and Cu(I) liquid crystal for advanced electrochemical detection of doxorubicine in aqueous solutions. Nanomaterials.

[40] Konopka S.J., McDuffie B. (1970). Diffusion coefficients of ferri- and ferrocyanide ions in aqueous media, using twin-electrode thin-layer electrochemistry. Anal. Chem..

[41] Negrea S., Diaconu L.A., Nicorescu V., Motoc S., Orha C., Manea F. (2021). Graphene oxide electroreduced onto boron-doped diamond and electrodecorated with silver (Ag/GO/BDD) electrode for tetracycline detection in aqueous solution. Nanomaterials.

[42] Mostofizadeh A., Li Y., Song B., Huang Y. (2011). Synthesis, properties, and applications of low-dimensional carbon-related nanomaterials. J. Nanomater..

[43] Zhang J., Lee J.-K., Wu Y., Murray R.W. (2003). Photoluminescence and electronic interaction of anthracene derivatives adsorbed on sidewalls of single-walled carbon nanotubes. Nano Lett..

[44] Honakeri N.C., Malode S.J., Kulkarni R.M., Shetti N.P. (2020). Electrochemical behavior of diclofenac sodium at coreshell nanostructure modified electrode and its analysis in human urine and pharmaceutical samples. Sens. Int..

[45] Cid-Ceron M.M., Guzman-Hernzandez D.S., Ramirez-Silva M.T., Galano A., Romero-Romo M., Palomar-Pardave M. (2016). New insigths on the kinetics and me-chanism of the electrochemical oxidation of diclofenac in neutral aqueous medium. Electrochim. Acta.

[46] Sanecki P., Skitał P., Kaczmarsk K. (2006). Numerical modeling of ECE-ECE and parallel EE-EE mechanisms in cyclic voltammetry. reduction of 1,4-benzenedisulfonyl difluoride and 1,4-naphthalenedisulfonyl difluoride. Electroanalysis.

[47] Goyal R.N., Chatterjee S., Agrawal B. (2010). Electrochemical investigations of diclofenac at edge plane pyrolytic graphite electrode and its determination in human urine. Sens. Actuators B Chem..

[48] Aguilar-Lira G.Y., Alvarez-Romero G.A., Zamora-Suarez A., Palomar-Pardave M., Rojas-Hernández A., Rodriguez-Avila J.A., Paez-Hernandez M.E. (2017). New insights on diclofenac electrochemistry using graphite as working electrode. J. Electroanal. Chem..

[49] Sasal A., Tyszczuk-Rotko K., Wojciak M., Sowa I. (2020). First electrochemical sensor (screen-printed carbon electrode modified with carboxyl functionalized multiwalled carbon nanotubes) for ultratrace determination of diclofenac. Materials.

[50] Mokhtari A., Karimi-Maleh H., Ensafi A.A., Beitollahi H. (2012). Application of modified multiwall carbon nanotubes paste electrode for simultaneous voltammetric determination of morphine and diclofenac in biological and pharmaceutical samples. Sens. Actuators B Chem..

[51] Okoth O.K., Yan K., Liu L., Zhang J. (2015). Simultaneous electrochemical determination of paracetamol and diclofenac based on poly(diallyldimethylammonium chloride) functionalized graphene. Electroanalysis.

