# Supplementary material for: Electrochemical Method for Ease Determination of Sodium Diclofenac Trace Levels in Water Using Graphene—Multi-Walled Carbon Nanotubes Paste Electrode
Source: Int J Environ Res Public Health. 2021 Dec 21;19(1):29. doi: 10.3390/ijerph19010029 (PMC8750901; doi:10.3390/ijerph19010029)
Supplement: Supplementary file 1 [file ijerph-19-00029-s001.zip › ijerph-1475144-supplementary.pdf]

International Journal of
Environmental Research and Public Health

MDPI

Supplementary material

Electrochemical Method for Ease Determination of Sodium Diclofenac Trace Levels in Water Using Graphene—Multi-Walled Carbon Nanotubes Paste Electrode

Sorina Motoc (m. Ilies) [1], Florica Manea [2,*], Anamaria Baciu [2], Corina Orha [3] and Aniela Pop [2]

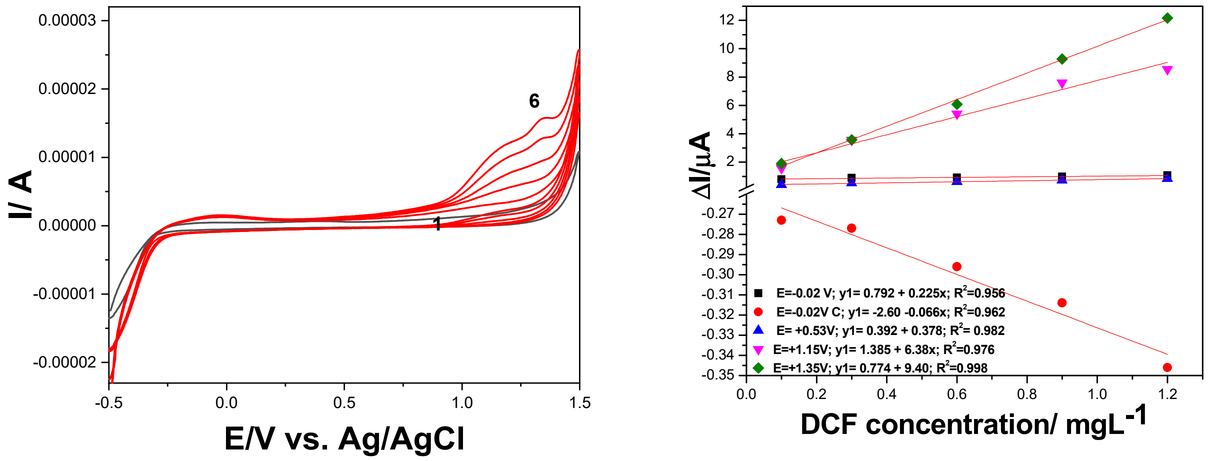

a) b)

Figure S1. a) CVs recorded at GR-CNT paste electrode with the scan rate of 0.050 $V \cdot s^{-1}$ in 0.100 M Na_2SO_4 supporting electrolyte (curve 1) and DCF concentrations ranged from 0.100 to 1.20 mgL^{-1} (curve 2-6). b) Calibrations plots of peak current vs DCF concentrations at the potential value: E = -0.050 V vs Ag/AgCl (anodic), E = +0.580 V vs Ag/AgCl (anodic), E = +1.180 V vs Ag/AgCl (anodic), E= +1.350 V vs Ag/AgCl (anodic) and E = -0.200 V vs Ag/AgCl (cathodic).

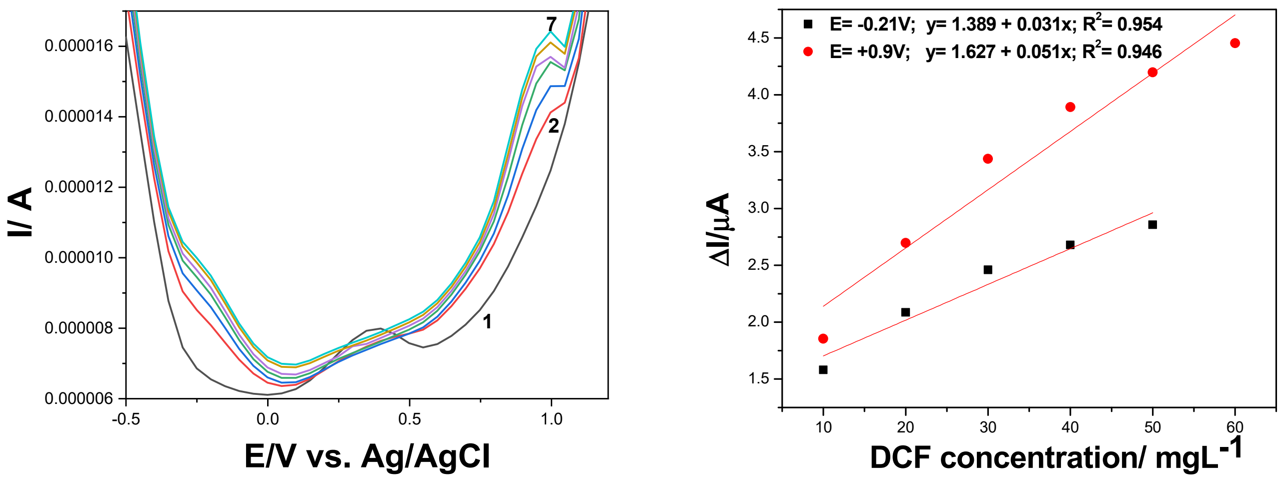

a) b)

Figure S2. a) Differential-pulsed voltammograms recorded on GR-CNT paste electrode in 0.100 M Na_2SO_4 supporting electrolyte (curve 1) with optimum accumulation time of 25 minutes in the presence of different DCF concentrations: 10-60 ng·L^{-1} (curves 2-7); step potential of 50 mV, modulation amplitude of 200 mV, and a scan rate of 100 mV·s^{-1}, potential range: -0.500 V to +1.500 V vs Ag/AgCl; b) Calibrations plots of peak current vs DCF concentrations at the potential value: E = -0.210 V vs Ag/AgCl , E = +0.900 V vs Ag/AgCl
